# Systematic investigation of promoter substitutions resulting from somatic intrachromosomal structural alterations in diverse human cancers

**DOI:** 10.1038/s41598-020-74420-2

**Published:** 2020-10-23

**Authors:** Babak Alaei-Mahabadi, Kerryn Elliott, Erik Larsson

**Affiliations:** Department Of Medical Biochemistry and Cell Biology, Institute of Biomedicine, The Sahlgrenska Academy, University of Gothenburg, 405 30 Gothenburg, Sweden

**Keywords:** Cancer, Computational biology and bioinformatics, Cancer genomics, Gene expression, Genomics

## Abstract

One of the ways in which genes can become activated in tumors is by somatic structural genomic rearrangements leading to promoter swapping events, typically in the context of gene fusions that cause a weak promoter to be substituted for a strong promoter. While identifiable by whole genome sequencing, limited availability of this type of data has prohibited comprehensive study of the phenomenon. Here, we leveraged the fact that copy number alterations (CNAs) arise as a result of structural alterations in DNA, and that they may therefore be informative of gene rearrangements, to pinpoint recurrent promoter swapping at a previously intractable scale. CNA data from nearly 9500 human tumors was combined with transcriptomic sequencing data to identify several cases of recurrent activating intrachromosomal promoter substitution events, either involving proper gene fusions or juxtaposition of strong promoters to gene upstream regions. Our computational screen demonstrates that a combination of CNA and expression data can be useful for identifying novel fusion events with potential driver roles in large cancer cohorts.

## Introduction

Copy number alterations (CNAs) significantly contribute to cancer development, usually by causing oncogene amplification or tumor suppressor deletion^1–3^. Well-characterized examples of cancer driver events involving CNAs are *CDKN2A*^4^ and *PTEN*^5^ deletions or *MYC*^6^, *EGFR*^7^ and *ERBB2*^2,7^ amplifications. With the availability of high-resolution SNP arrays, several studies have comprehensively investigated these events in cancer, mainly focusing on gene amplitude changes^8,9^.

CNAs are a consequence of changes in chromosome structure^10^. CNAs may therefore be indicative of more complex rearrangements of genomic features such as regulatory elements that determine the transcriptional activity of genes. Recent studies have indeed uncovered that deletions and duplications may facilitate mRNA level changes by shuffling or amplifying non-coding regions in the genome including *cis*-regulatory elements such as enhancers^11–14^. Another known mechanism for a gene to be activated by genomic structural variations (SVs) is to substitute its promoter with a stronger promoter in the context of gene fusions^15,16^. One of the most frequent promoter substitution (PS) events in cancer involve transcriptional activation of *ERG* through fusion with *TMPRSS2*, which occurs in approximately 40% of prostate cancers as a result of a genomic deletion on chromosome 17q22^17^. Several other fusions involving this mechanism are known^18–20^. Furthermore, in a recent study based on whole genome sequencing (WGS) data from 600 tumors, we observed several non-recurrent cases of PS that arose due to intrachromosomal SVs, specifically deletions or inversions, which were associated with transcriptional activation^21^. Investigations based on larger cohorts could potentially give insights into whether or not such events are recurrent, suggestive of positive selection and thereby importance in cancer.

In this study, we used 9423 array-based copy number profiles made available by The Cancer Genome Atlas consortium to identify deletions and likely tandem duplications predicted to result in intrachromosomal PS events, due to either proper gene fusions or juxtaposition of strong promoters to upstream regions. We then investigated the relationship between such events and mRNA level changes. By using CNAs as a proxy of SVs, we could thus investigate this phenomenon in a cohort that is considerably larger than what is currently possible using WGS.

## Results

### Mapping of tandem duplications and deletions using copy number profiles in a large cancer cohort

**Figure 1:**
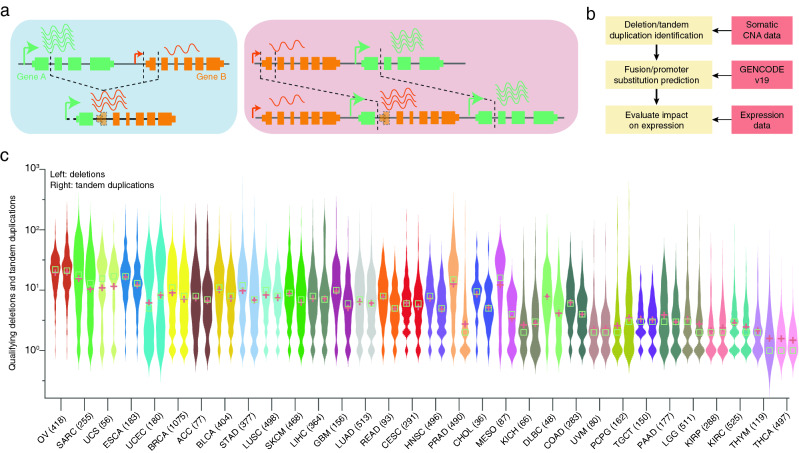
Pipeline overview. (**a**) Underlying principle of the promoter substitution events. A deletion, shown in the blue box and a tandem duplication, shown in the red box resulting in the substitution of the strong promoter of green gene A with the weaker promoter of orange gene B. The breakpoints near orange gene B could be both within and upstream of the gene body. (**b**) Analysis workflow: 9423 SNP6 derived copy number profiles and RNA-seq based gene expression profiles were used in this study. (**c**) Violin plot showing SNP6 based deletions (left) and tandem duplications across (right) multiple cancer types. OV, Ovarian serous cystadenocarcinoma; SARC, Sarcoma; UCS, Uterine Carcinosarcoma; ESCA Esophageal carcinoma; UCEC, Uterine Corpus Endometrial Carcinoma; BRCA, Breast invasive carcinoma; ACC, Adrenocortical carcinoma; BLCA, Bladder Urothelial Carcinoma; STAD, Stomach adenocarcinoma; LUSC, Lung squamous cell carcinoma; SKCM, Skin Cutaneous Melanoma; LIHC, Liver hepatocellular carcinoma; GBM, Glioblastoma multiforme; LUAD, Lung adenocarcinoma; READ, Rectum adenocarcinoma; CESC, Cervical squamous cell carcinoma and endocervical adenocarcinoma; HNSC, Head and Neck squamous cell carcinoma; PRAD, Prostate adenocarcinoma; CHOL, Cholangiocarcinoma; MESO, Mesothelioma; KICH, Kidney Chromophobe; DLBC, Lymphoid Neoplasm Diffuse Large B-cell Lymphoma; COAD, Colon adenocarcinoma; UVM, Uveal Melanoma; PCPG, Pheochromocytoma and Paraganglioma; TGCT, Testicular Germ Cell Tumors; PAAD, Pancreatic adenocarcinoma; LGG, Brain Lower Grade Glioma; KIRP, Kidney renal papillary cell carcinoma; KIRC, Kidney renal clear cell carcinoma; THYM, Thymoma; THCA, Thyroid carcinoma. Colors in this graph are used throughout to indicate cancer type. The distribution of deletions and tandem duplications is shown for each cancer type. Green boxes and red plus signs show the median and mean, respectively.

With the ultimate aim of detecting PS events resulting from intrachromosomal SVs in a large multi-cancer cohort (Fig. 1a), we first sought to identify somatic tandem duplications and deletions using Genome-Wide Human SNP Array 6.0 (SNP6) data from The Cancer Genome Atlas (TCGA; Fig. 1b). The probe-based nature of this data limits its resolution and it is also affected by sample purity and ploidy, and we therefore applied strict filtering criteria to ensure that only events with a clear interpretation in terms of the structural basis were considered (see Methods). By comparing with WGS-based SVs from a previous study^21^, available for a subset of samples (600 tumors), we found that 25% of the CNA-inferred SVs had a correspondence in WGS-based SVs and of these, 97% were coherently classified as deletions or duplications in the two datasets.

In the complete cohort, comprising of 9423 tumors from 32 different cancer types, we identified 110,463 predicted deletions and 84,052 tandem duplications that fulfilled our criteria. The number of events varied considerably between cancer types, with the highest (OV, SARC) and lowest (THYM, THCA) numbers seen in cancers previously shown to have many or few SVs based on analysis of WGS data (Fig. 1c)^21^. The number of deletions and tandem duplications were typically comparable in a given cancer type (both plots within twofold; Fig. 1c). However, two cancer types, prostate (PRAD) and mesothelioma (MESO), had elevated number of deletions relative to duplications (4.2 and 5.2-fold difference, respectively).

### Pan-cancer analysis of SVs resulting in promoter substitutions

**Figure 2:**
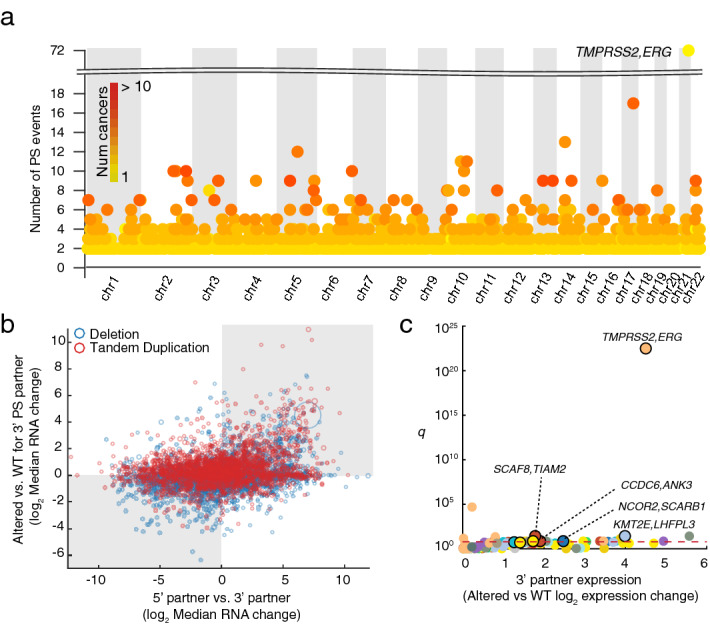
Pan-cancer analysis of SVs resulting in promoter substitutions. (**a**) Manhattan plot showing the recurrent promoter substitution (PS) events across the genome by chromosome in all cancer types. Note that the dot color represents the number of cancer types with the event. (**b**) Induction of 3′ partners tends to occur when the 5′ partners have a stronger promoter. The log2 transformed expression difference of 3′ partner of PS events, comparing affected samples with the median of the unaffected samples in the same cancer type is shown on Y-axis. X-axis represents the expression difference of 5′ partner with the 3′ partner, comparing median expression within the same cancer type. Circle sizes correspond to the frequency of the event. (**c**) Volcano plots showing recurrent cases with 3′ partner induction, where the 5′ partner has the stronger promoter. Pairs highlighted in the text are labeled. Cancers are color-coded similar to Fig. 1c. WT, unaffected wild type samples, with respect to the indicated alteration, from the same cancer type. q, false discovery rate.

We next identified a subset of SVs that may result in PS (Methods), involving either gene fusions or alternatively cases where the 5′ promoter is juxtaposed to the upstream region of the 3′ partner (Fig. 1a). We found 20,715 SVs having PS potential comprising of 9754 tandem duplications and 10,961 deletions. 1925 unique gene pairs were involved in recurrent (*n* > 1) predicted PS events (Fig. 2a). Confirming previous reports, the most recurrent case was *TMPRSS2*-*ERG* (*n* = 72; Supplementary Fig. S1a), which was completely restricted to prostate cancer (*n* = 490 samples). The observed frequency was lower than previously reported^17^, and therefore we manually explored copy number profiles for the complete prostate cancer cohort, which revealed 28 additional cases with deletions potentially fusing these two genes. These were not detected by our pipeline due to presence of more complex copy number patterns in the region (Supplementary Fig. S2). A subset of 20 prostate samples had available WGS data and four of these were previously found to harbor the *TMPRSS2*-*ERG* fusion^21^, all of which were confirmed using the copy number pipeline. Notably, while many known functional fusions, including *TMPRSS2*-*ERG,* are restricted to specific cancer types, we observed that most recurrent cases were distributed across multiple cancers (Fig. 2a). While this does not exclude that they could be functional, further analysis was motivated.

We next investigated associations between recurrently PS affected cases and gene expression changes. As expected, we found that mRNA expression of the 3′ partner increased when the 5′ partner had a stronger promoter, the latter determined by comparing the median expression levels of the two partners in a given cancer type (Fig. 2b; *p* = 2.51e−22, Fisher’s exact test). Additionally, we found that transcriptional induction of the 3′ partner occurred more frequently when the genes were closer together (Supplementary Fig. S3). There were 126 cases of genes being recurrently (*n* >  = 2) affected by PS with a stronger promoter (2-fold) within an individual cancer type. In 8 of these cases, the 3′ partner gene was significantly induced in PS-affected samples (Student’s *t-*test at FDR 10%; Fig. 2c), although it should be noted that the statistical power was weak due to number of affected samples typically being small.

The most significant case was *TMPRSS2*-*ERG* with 22-fold increase in expression (*p* = 2.56e−25 uncorrected; Supplementary Fig. S1b). We also identified the previously reported *ESR1*-*CCDC170* fusion^22^ in breast cancer (*n* = 3), associated with *CCDC170* elevated expression resulting from recruiting the strong promoter of *ESR1* (*p* = 0.02; Supplementary Fig. S4). One additional ovarian tumor harbored the same fusion, although induction of *CCDC170* was not significant (*p* = 0.44).

### Novel recurrent promoter substitution events

**Figure 3:**
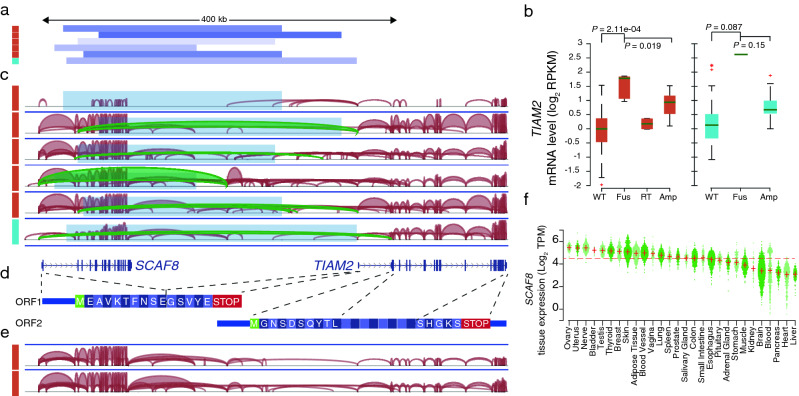
TIAM2 overexpression as a result of promoter substitution with *SCAF8*. (**a**) Genomic deletions (blue bars) juxtapose the *SCAF8* promoter (below Fig. 3c) in five ovarian (brown) and one endometrial (cyan) tumors. Cancers are color-coded similar to Fig. 1c. (**b**) Strong activation of *TIAM2* in PS positive cases. mRNA level of *TIAM2* is shown across 418 ovarian and 180 endometrial tumors. Wild type tumors (WT) without *SCAF8*-*TIAM2* fusions, *SCAF8*-*TIAM2* fusion (Fus), as well as two *SCAF8*-*TIAM2* read through (RT) and amplified samples (Amp) are shown separately in ovarian cancer samples (brown). *P*-values are calculated using the Wilcoxon rank-sum test comparing the expression of the altered tumors with other samples. (**c**) Splice junction derived from RNA-Seq data for PS events. Red arcs are RNA reads. Reads supporting the deletions (blue boxes) are shown in green. (**d**) Two possible ORFs of the new fusion transcript. Blue lines indicate exon structure for *SCAF8* and *TIAM2* genes. Dashed lines show exon junctions. (**e**) Splice junction for two samples with read through events are shown here. (**f**) *SCAF8* expression across normal tissues from GTEx. Red plus signs indicate mean expression per tissue. The dashed red line indicates the mean expression of all tissue samples.

Notable among novel significant cases was predicted fusions between *SCAF8* and *TIAM2* resulting from deletions bridging these two closely positioned neighbor genes (Fig. 3a). This occurred primarily in ovarian carcinoma (*n* = 5), specifically in the serous histological subtype, where *TIAM2* expression was increased 3.4-fold in PS-affected cases compared to remaining samples (*p* = 8.71e−04 uncorrected), and was also found in endometrial carcinoma (*n* = 1; 5.5-fold; *p* = 0.086 uncorrected). *TIAM2* acts as an upstream regulator in the Rac pathway, and it has been shown that the overexpression of this gene promotes cell proliferation and invasion in multiple cancer types^23–25^. Interestingly, induction of *TIAM2* in PS positive cases surpassed what was seen in cases of *TIAM2* gene amplification (Fig. 3b).

We next focused on understanding the transcript and protein structure resulting from these deletions. We found that in 5/6 cases with RNA level support, the first or second exon of *SCAF8* was fused with the first noncoding exon of *TIAM2* located in the 5′ UTR (Fig. 3c). This resulted in a novel transcript containing a smaller truncated open reading frame (ORF) from *SCAF8* followed by the complete *TIAM2* mRNA sequence including the 5′ UTR, thereby containing two possible ORFs (Fig. 3d). More work is needed to determine whether TIAM2 can be translated from this transcript.

Analysis of RNA-Seq data from all included ovarian and endometrial tumors revealed two additional samples with *SCAF8*-*TIAM2* fusion transcripts in the absence of DNA-level support, suggesting that this could be due to read through events (Fig. 3e). Notably, *TIAM2* induction was considerably lower in these cases compared to those with genomic deletions (Fig. 3b). Based on the GTEx panel of human tissues, we found that wild type *SCAF8* had its highest expression in the ovary and uterus, making it an ideal fusion partner to drive high expression in ovarian and endometrial cancers (Fig. 3f). Additionally, we found that deletion breakpoints did not overlap with common fragile sites in the HumCFS database^26^. This, together with the tissue-restricted pattern, further supported that the reported events may be due to positive selection specifically in these cancers.

**Figure 4:**
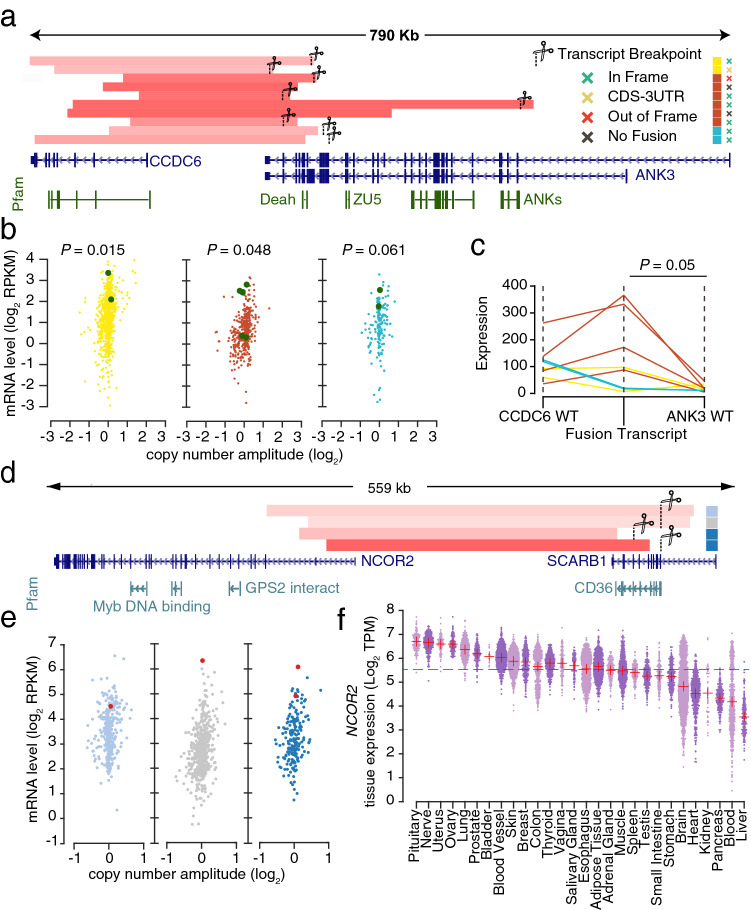
Overexpression of ANK3 and SCARB1 through hijacking the strong promoter of CCDC6 and NCOR2. (**a**) Recurrent tandem duplication events causing *CCDC6*-*ANK3* fusion. Red bars indicate copy number gains, blue lines indicate exon structure of genes, green lines indicate Pfam protein domains. The scissors show the transcript junction for ANK3 derived from matching RNA-Seq. Cancers are color-coded similar to Fig. 1c. Crosses next to cancer types indicate how the transcript breakpoint affects the coding sequence (CDS) (**b**) Expression versus copy number change for *ANK3* in breast (yellow), ovarian (brown) and endometrial (cyan) respectively. PS positive samples are marked in green. *P* values are calculated using the Wilcoxon rank-sum test comparing the expression of the altered tumors with other samples. (**c**) The novel fusion transcript is expressed at a higher level compared to the WT *ANK3*. Read count based estimation of the expression level of the WT 5′ gene (*CCDC6*), the WT 3′ gene (*ANK3*) and the predicted chimeric gene (*CCDC6*-*ANK3*) was calculated using ericScript tool. (**d**) Recurrent tandem duplications creating a novel transcript containing the two first noncoding exons of *NCOR2* and *SCARB1*. Red bars indicate amplified regions, blue lines indicate exon structure of genes, grey lines indicate Pfam protein domains. (**e**) Expression versus copy number change for *SCARB1* in stomach (light blue), lung adenocarcinoma (light grey) and esophageal (dark blue) respectively. PS positive samples are marked in red. (**f**) *NCOR2* expression across different tissues from GTEx. Red plus signs indicate mean expression per tissue. The dashed red line indicates the mean expression of all tissue samples. TPM, transcripts per million.

Novel recurrent cases were also found in ovarian (*n* = 6), endometrial (*n* = 2) and breast (*n* = 2) cancers involving *CCDC6*, a coiled-coil domain protein, fusing with *ANK3* at the 3′ end, which encodes the ankyrin G protein that plays a key role in cell proliferation, as result of tandem duplications (Fig. 4a). While it has been shown that downregulation of *ANK3* is associated with poor prognosis in multiple cancers such as prostate, ovarian, lung and breast^27^, a recent study also described that increased *ANK3* contributes to prostate cancer progression, implying that both up and down regulation of this gene can be important at different clinical stages^28^. Here, we observed that fusion with *CCDC6* was associated with strong overexpression of *ANK3* in all three cancers (Fig. 4b). In 6/10 cases the regulatory domain of *ANK3*, also known as death like domain^29^, was retained. Furthermore, we observed that *CCDC6* is normally highly expressed in ovarian, endometrial and breast compared to the other cancer types, as well as in normal ovary and uterine tissues (Supplementary Fig. S5). Further analysis of matching RNA-Seq samples showed that the fusion transcript was significantly upregulated compared to the wild type *ANK3* form, consistent with *ANK3* gaining the strong promoter from *CCDC6* (Fig. 4c).

Another recurrent case (*n* = 4) was found in stomach, esophageal and lung adenocarcinoma, where *SCARB1*, a high-density lipoprotein (HDL) receptor, was overexpressed through fusion with *NCOR2* due to tandem duplications on chromosome 12q24 (Fig. 4d,e). Notably, the functionally critical CD36 family domain of *SCARB1*, a receptor family that is crucial for cholesterol uptake, was maintained in all cases. Consistent with the elevated expression of *SCARB1*, we found that the *NCOR2* gene is relatively highly expressed in the relevant tissue types, making it a suitable 5′ partner for activating transcription (Fig. 4f). Overexpression of *SCARB1* has been associated with cancer development and shown to be inversely correlated with survival in multiple cancer types, although no molecular mechanism was proposed^30,31^.

Finally, we observed overexpression of *LHFPL3* in four stomach tumors harboring *KMT2E*-*LHFPL3* fusions arising due to tandem duplications on chromosome 7q22 (Supplementary Fig. S6a,b). Interestingly, in three of the four cases, a valid fusion transcript was supported by RNA-Seq, expressed at elevated levels compared to the *LHFPL3* unaltered transcript (Supplementary Fig. S6c). Although more work is needed to determine the relevance of these events, it can be noted that *LHFPL3* is a member of the LHPF-like gene family known to be fusion partners of *HMGIC*, an established tumor associated gene in lipoma^32^, and overexpression of this gene has been described in ovarian cancer^33^.

Investigation of the PS events described above in copy number profiles from the Cancer Cell Line Encyclopedia (CCLE) database confirmed all cases except *TIAM2*-*SCAF8* (Supplementary Fig. S7a). An amplification predicted to form a *NCOR2*-*SCARB1* fusion gene was identified in one lung cancer cell line (Supplementary Fig. S7b), *CCDC6*-*ANK3*-forming amplifications were found in two ovarian cancer cell lines (Supplementary Fig. S7c), and a *LHFPL3*-*KMT2E*-forming amplification was found in one lung cancer (Supplementary Fig. S7d). The known fusion *CCDC170*-*ESR1* was found in three breast cancer samples (Supplementary Fig. S7e) while the well-described promoter substitution event, *TMPRSS2*-*ERG* (Supplementary Fig. S7f), was identified in one prostate cancer cell line.

## Discussion

Promoter substitutions, whereby structural genomic changes lead to one gene gaining a promoter from another gene, is a known mechanism for transcriptional activation of oncogenes in cancer^34,35^, but the phenomenon has not previously been comprehensively investigated. Here, we took advantage of the fact the CNA profiles gives insight into structural genomic alterations, which, when combined with expression data, enabled mapping of putative PS events in a large multi-cancer cohort. CNA data have several limitations in this context, including not being informative about inversions and interchromosomal SVs. Furthermore, the array-based CNA data used in this study has limited resolution, and sensitivity may be reduced in some samples with lower sample purity. However, in return there is abundant availability of CNA profiles from human tumors, enabling detection of events that are recurrent at frequencies that are undetectable in WGS-based analysis. While only ~ 25% of CNA-based events were confirmed using WGS-based SV analysis, to a large extent this is likely to reflect of the limited the sensitivity of WGS-based SV data, and events detected using both datatypes showed a high degree of consistency (97%) in terms of deletion/duplication classification. Importantly, using our combined CNA and expression approach, we confirm several established cases and also identify new cases of recurrent PS.

The *TIAM* gene family is part of the Rac signaling pathway, and has been shown to contribute to tumor development in multiple cancer types^36–38^. Genomic alterations involving the *TIAM1* gene have been previously described^39,40^, and *TIAM2* has been shown to be upregulated in lung and liver tumors^23,25^, but little is known about the underlying mechanism for this activation. Here, we describe a novel mechanism leading to *TIAM2* overexpression in ovarian and endometrial carcinoma, that involves formation of a new fusion transcript transcribed from a nearby promoter that is highly active in these tissue types. More work is needed to determine if the resulting mRNA, which has an unusual structure, can serve as a template for *TIAM2* translation, but the fact that the transcript is abundant suggests avoidance of nonsense-mediated decay and hence proper translation. The functional consequences of increased TIAM2 protein levels in these tumor types will need to be determined in future experimental studies.

Several studies have shown that the cholesterol plays a role in development of cancer^41,42^. *SCARB1* is a protein that is involved in transporting HDL cholesterol in the body, and overexpression of this gene is known to facilitate this mechanism. Although the activation of *SCARB1* has been shown to be associated with tumor size and worse overall survival in cancer^43^, the underlying mechanism by which this gene becomes active is poorly understood. Here, we show that PS can activate *SCARB1* in three cancer types, although more work is needed to determine whether these are driving events.

In summary, we leveraged CNA and expression profiles available for nearly 10,000 tumors to screen for cases where genes were transcriptionally activated due to fusion with nearby genes having strong promoters, pinpointing several events with potential importance for cancer development. While the extent to which these events are due to positive selection remains an open question, it should be noted that they occur recurrently, sometimes in a tissue-restricted manner, and affect genes previously implicated in cancer. Future experimental studies should aim to investigate the functional consequences of these events in cancer.

## Methods

### Copy number and gene expression data processing

SNP6 segmented copy number profiles from 9423 tumors in 32 cancer types were obtained from the TCGA data portal. We classified segments into 5 copy number state categories in regards to their log_2_ amplitude provided in the raw seg files. Segments with seg_mean < − 1 were classified in homozygous deletions, − 1 < = seg_mean < − 0.2 hemizygous deletions, − 0.2 < = seg_mean < − 0.3 neutral, 0.3 < = seg_mean < 0.7 gain and, seg_mean = > 0.7 amplifications. Nearby segments with the same copy number state were merged to a bigger segment. Segments adjacent to a no-data regions bigger that 100 kb were removed for further analysis.

SV deletions were defined as (1) hemizygous deleted region where neither of adjacent segments were homozygous deletions and (2) homozygous deleted segments where both adjacent segments were hemizygous deletions. Gained segments with no adjacent “amplified segments” were considered as SV tandem duplications. SVs with breakpoints within 2 Mb range of telomeres and centromeres, or smaller than 15 Kb were removed for further analysis. The breakpoints were annotated against GENCODE v19 gene annotation with the following priority: overlapping coding gene, overlapping lincRNA, and closest upstream gene.

Matching RNA-Seq data were downloaded from the TCGA portal and used to quantify gene expression as described previously^44^. Normal tissue expression was obtained from the GTEx portal. Fusion transcripts were detected using ericScript^45^. WGS-based SV data for a subset of samples (600) was obtained from Alaei-Mahabadi, et al.^21^.

### Screening for association between SVs and RNA levels resulting from promoter switching

SVs resulting in a valid PS cases were identified using the following logic: We considered two different cases: (1) SVs predicted to produce a viable fusion between two genes, i.e. where both breakpoints fell within annotated genes and where both genes were transcribed in the same direction. In this case, the gene on the 3′ side will have gained the promoter from the 5′ partner gene. (2) SVs predicted to fuse the 5′ part of a gene (including the promoter) with a position somewhat upstream of another gene transcribed in the same direction. This may lead to the promoter of the 5′ partner gene driving expression of the 3′ partner due to transcriptional readthrough. In this case, the 3′ partner gene was required to be located no further than 200 kb downstream of the breakpoint. Only coding genes were considered in the analyses. Read count based estimation of the expression levels of the WT 5′ gene, the WT 3′ gene and the predicted chimeric gene was based on ericScript^45^. These values were used to visualize the transcriptional consequences of the predicted fusion events.

### Confirming fusions in cancer cell line encylopedia CNA data

In order to confirm the presence of fusions we obtained copy number profiles from the CCLE https://portals.broadinstitute.org/ccle. Fusion genes were identified and samples sorted by breakpoint frequency to identify samples with CNVs at the known fusion sites in Integrative Genomics Viewer (IGV).

## Supplementary information

Supplementary Figures.

## Data Availability

The datasets analysed during the current study are available here: TCGA: SNP Array 6.0, WGS and RNA-seq, https://portal.gdc.cancer.gov/, GTEx: Normal tissue expression, https://www.gtexportal.org/home/datasets, CCLE: Cancer Cell Line Encyclopedia, https://portals.broadinstitute.org/ccle.
