# Supplementary material for: Systematic investigation of promoter substitutions resulting from somatic intrachromosomal structural alterations in diverse human cancers
Source: Sci Rep. 2020 Oct 23;10:18176. doi: 10.1038/s41598-020-74420-2 (PMC7584658; doi:10.1038/s41598-020-74420-2)
Supplement: Supplementary file 1 — Supplementary Figures. [file 41598_2020_74420_MOESM1_ESM.pdf]

Supplementary figures

Systematic investigation of promoter substitutions resulting from somatic intrachromosomal structural alterations in diverse human cancers

Babak Alaei-mahabadi, Kerryn Elliott, Erik Larsson

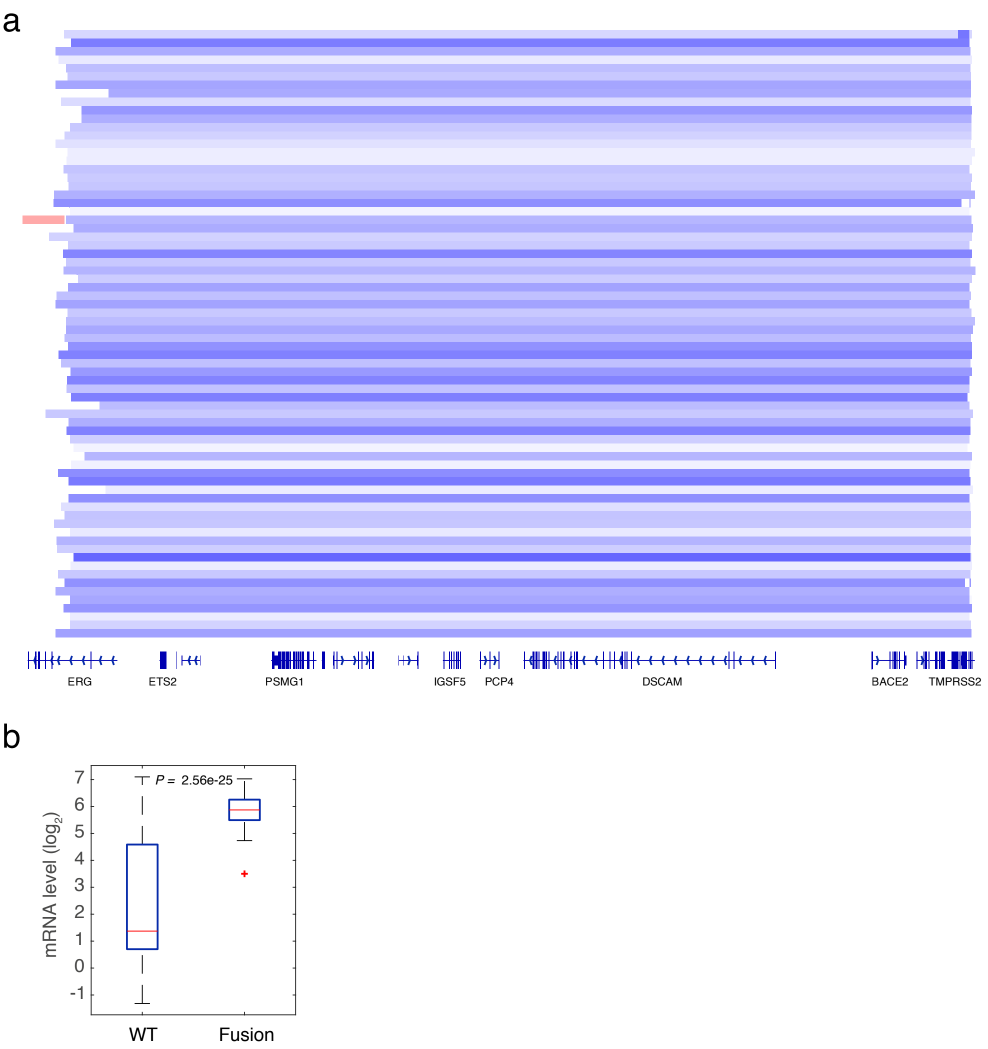

Supplementary Fig. S1. TMPRSS2-ERG fusion in 72 prostate cancers. (**a**) Genomic deletions (blue bars) were found in 72 tumors supporting the TMPRSS2-ERG fusion. The exon-intron structures of genes in the deleted region are shown at the bottom. (**b**) Cases with the fusion had significantly higher expression compared to the WT samples without the fusion. *P*-values are calculated using the Wilcoxon rank-sum test comparing the expression of the altered tumors with other samples.

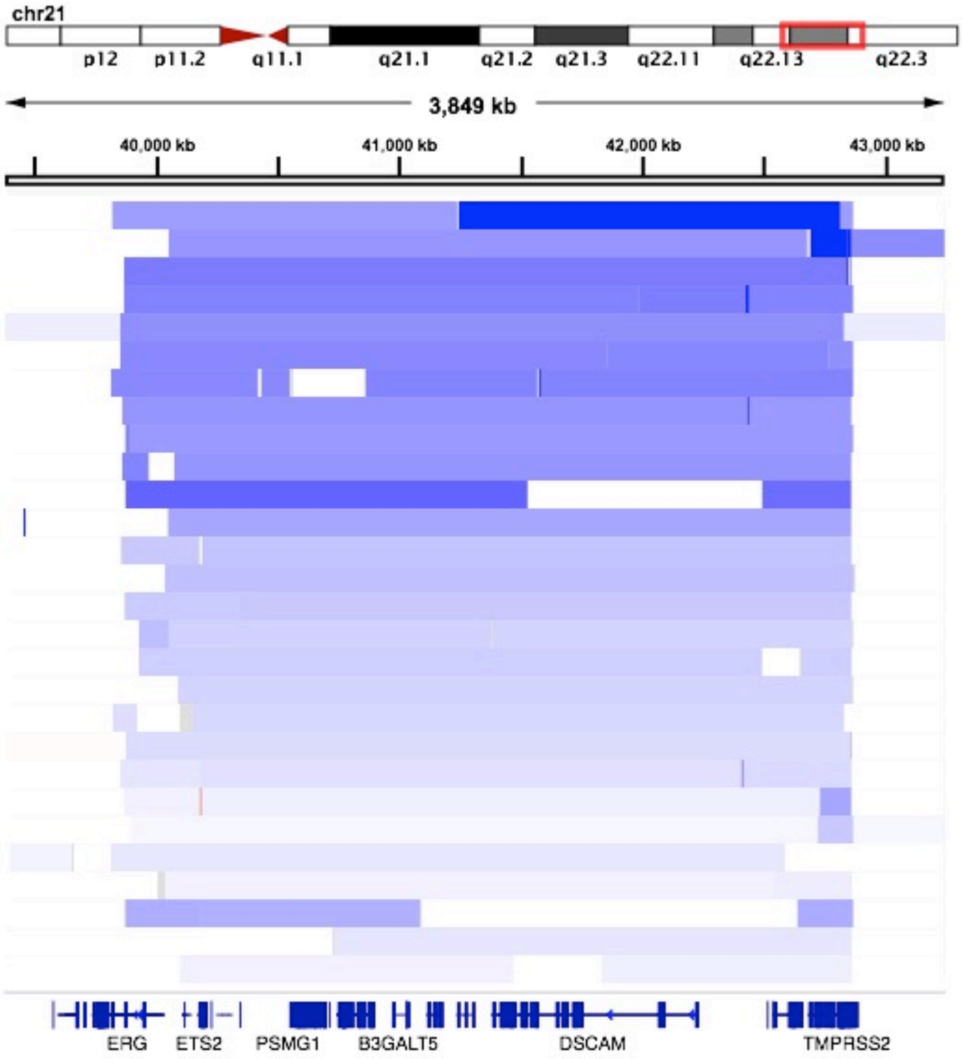

Supplementary Fig. S2. Additional samples with likely TMPRSS2-ERG fusion. Each row represents one tumor and blue bars represent copy number losses. The exon-intron structures of genes in the deleted region are shown at the bottom. Overlapping copy number events prevent the detection of the single events corresponding to this fusion.

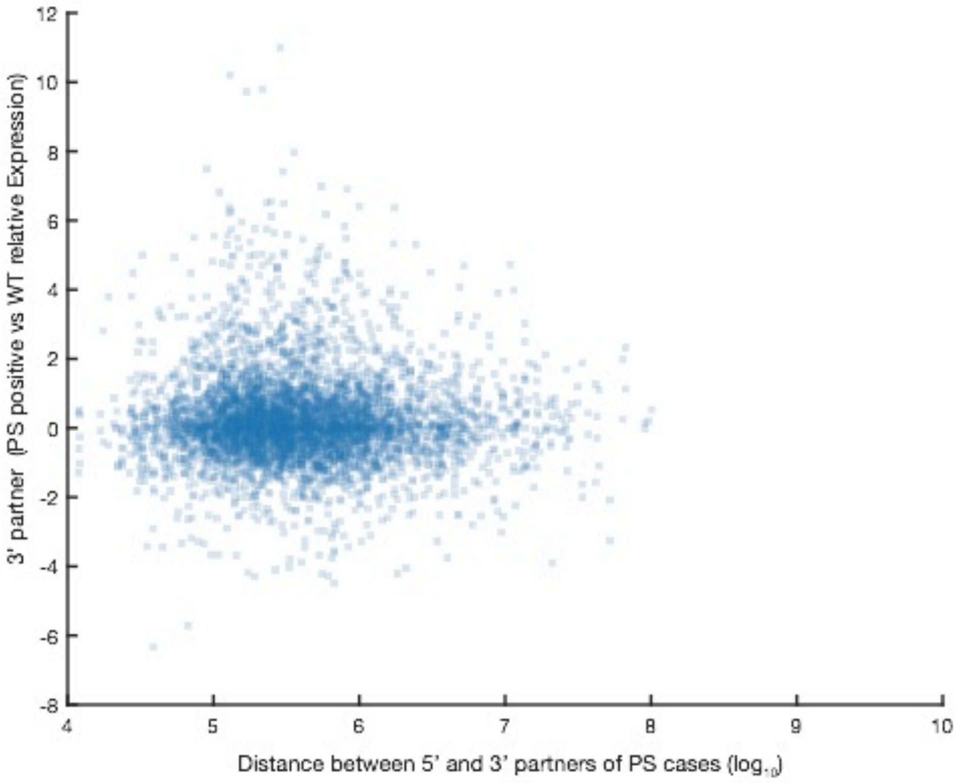

Supplementary Fig. S3. Overexpression of the 3' partner occurred more frequently when the fusion partners were more closely positioned in the genome.

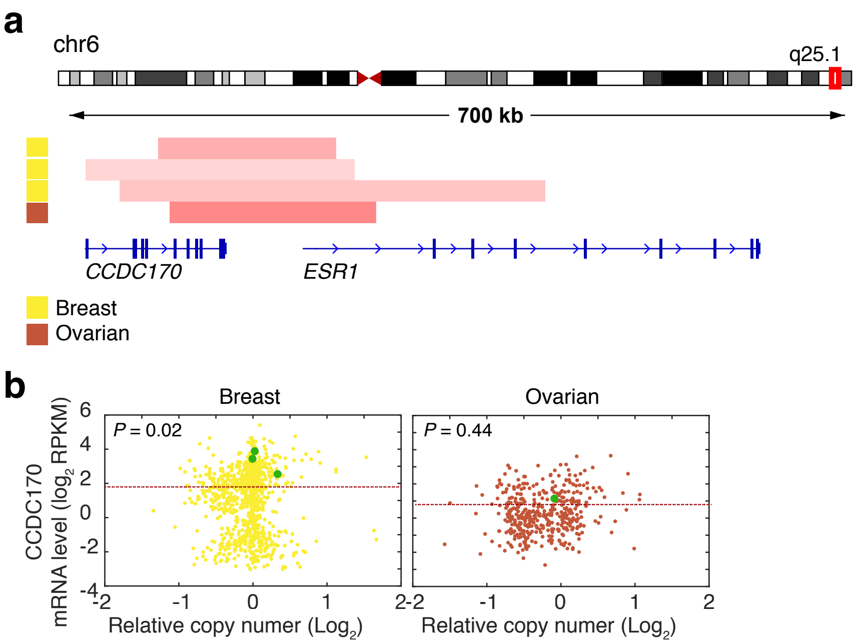

Supplementary Fig. S4. Induction of *CCDC170* by hijacking the strong promoter of *ESR1*. (**a**) Red bars represent copy number gains. The exon-intron structures of genes in the deleted region are shown at the bottom. (**b**) Expression vs. copy number change is shown for the two relevant cancer types breast.

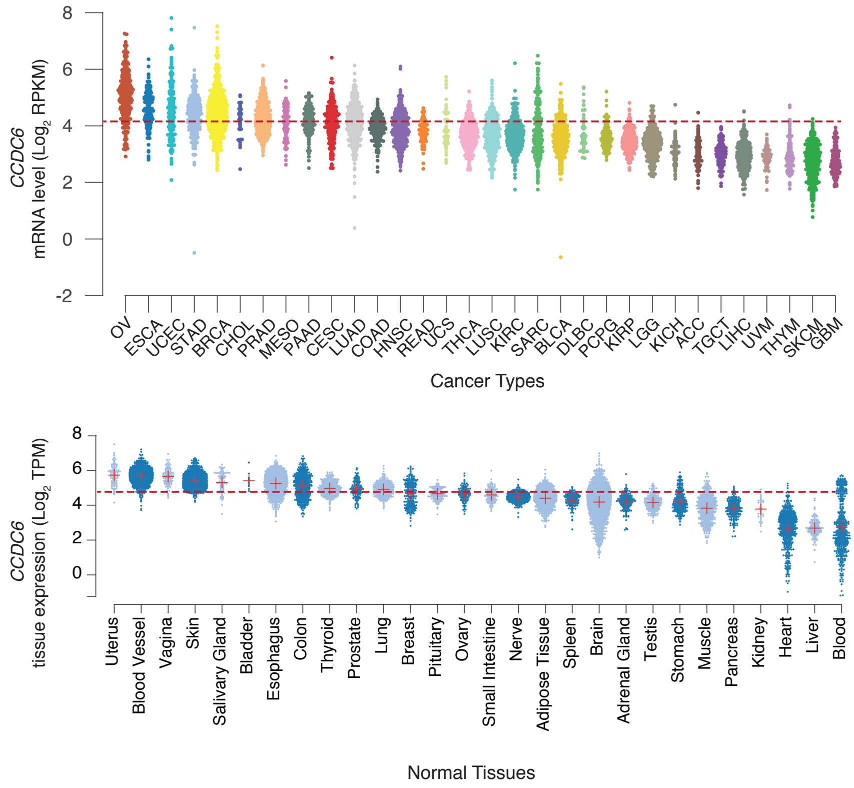

Supplementary Fig. S5. Expression of *CCDC6*. The plot above shows the mRNA level of *CCDC6* across multiple cancer types. OV, Ovarian serous cystadenocarcinoma; SARC, Sarcoma; UCS, Uterine Carcinosarcoma; ESCA Esophageal carcinoma; UCEC, Uterine Corpus Endometrial Carcinoma; BRCA, Breast invasive carcinoma; ACC, Adrenocortical carcinoma; BLCA, Bladder Urothelial Carcinoma; STAD, Stomach adenocarcinoma; LUSC, Lung squamous cell carcinoma; SKCM, Skin Cutaneous Melanoma; LIHC, Liver hepatocellular carcinoma; GBM, Glioblastoma multiforme; LUAD, Lung adenocarcinoma; READ, Rectum adenocarcinoma; CESC, Cervical squamous cell carcinoma and endocervical adenocarcinoma; HNSC, Head and Neck squamous cell carcinoma; PRAD, Prostate adenocarcinoma; CHOL, Cholangiocarcinoma; MESO, Mesothelioma; KICH, Kidney Chromophobe; DLBC, Lymphoid Neoplasm Diffuse Large B-cell Lymphoma; COAD, Colon adenocarcinoma; UVM, Uveal Melanoma; PCPG, Pheochromocytoma and Paraganglioma; TGCT, Testicular Germ Cell Tumors; PAAD, Pancreatic adenocarcinoma; LGG, Brain Lower Grade Glioma; KIRP, Kidney renal papillary cell carcinoma; KIRC, Kidney renal clear cell carcinoma; THYM, Thymoma; THCA, Thyroid carcinoma. The lower panel shows the expression of *CCDC6* across multiple normal tissue types from GTEX. The dashed red lines indicates the mean of all samples. TPM, transcripts per million.

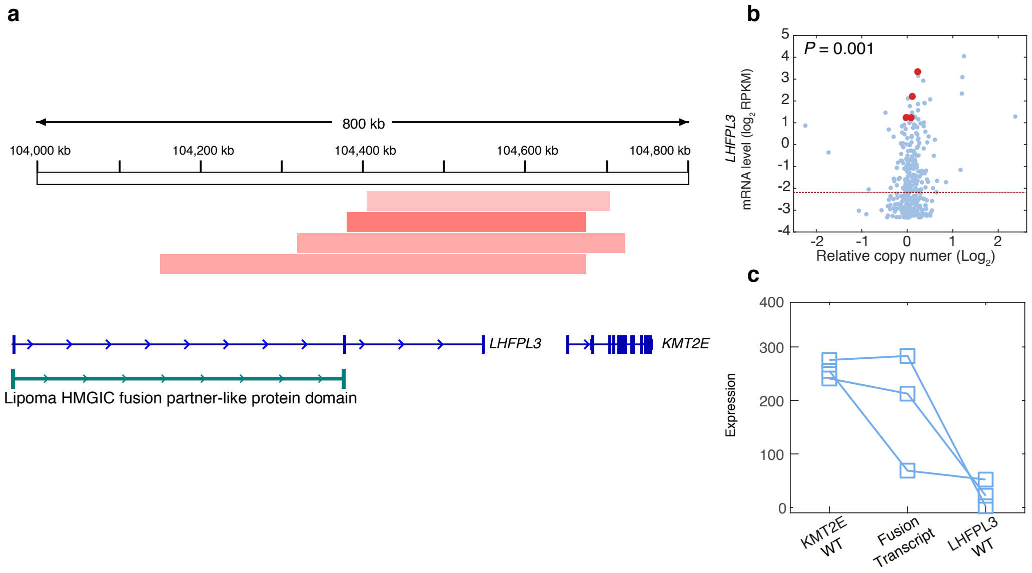

Supplementary Fig. S6. Induction of *LHFPL3* by hijacking the strong promoter of *KMT2E*. (**a**) Four stomach tumors with tandem duplications spanning these two adjacent genes. Red bars indicate copy number gains and green lines indicate Pfam protein domains. The exon-intron structures of genes in region are indicated in blue. (**b**) Expression vs. copy number change is shown for the complete stomach adenocarcinoma cohort. Red dots are samples shown in panel **a**. (**c**) The novel fusion transcript is expressed at higher level compared to the WT *LHFPL3*. Expression levels were quantified using the ericScript tool.

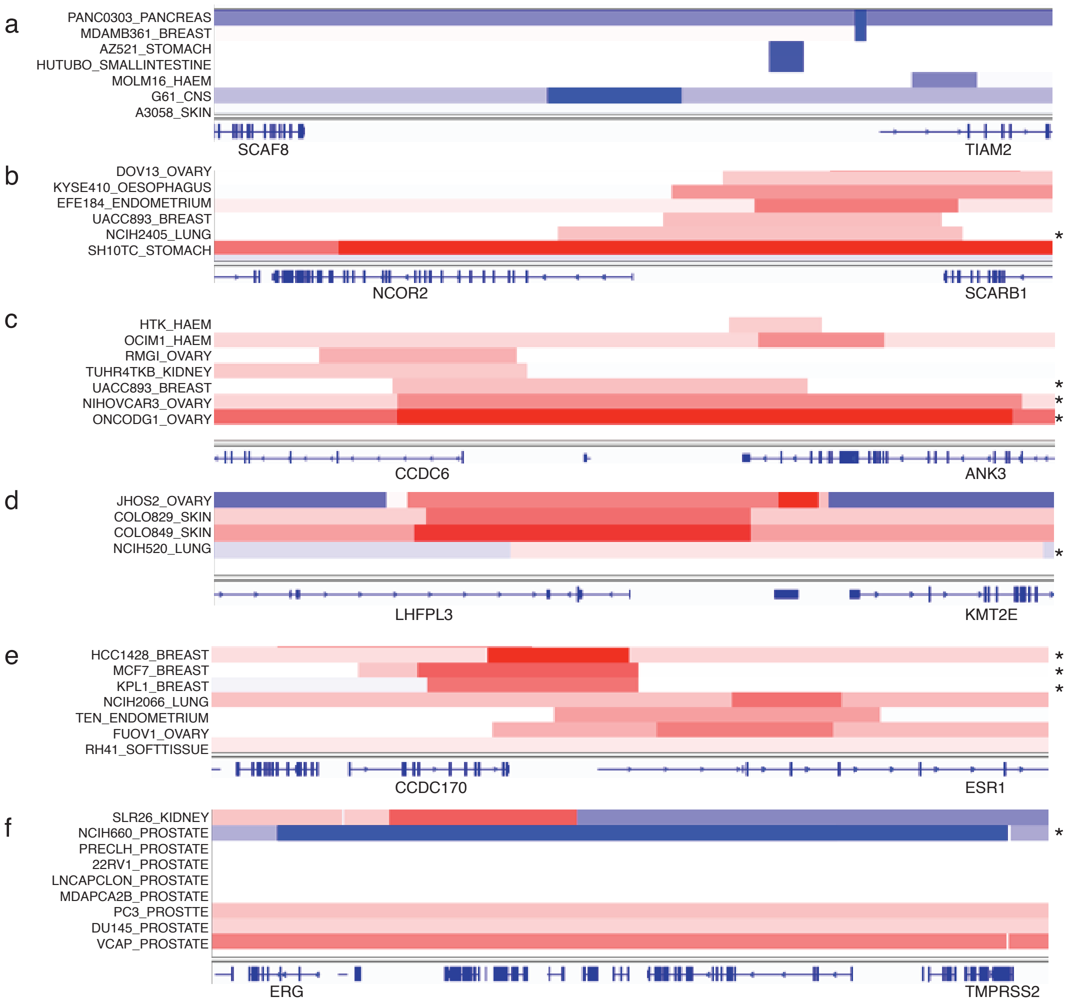

Supplementary Fig. S7. Analysis of fusion genes in CCLE. Reported fusion genes were analysed using copy number data available from the Cancer Cell Line Encylopedia for (**a**) *SCAF8-TIAM2* (**b**) *NCOR2-SCARB1* **(c)** *CCDC6-ANK3* (**d**) *LHFPL3-KMT2E* (**e**) *CCDC170-ESR1* and (**f**) *TMPRSS2-ERG*. Red bars indicate copy number gains and blue bars copy number losses. Cell lines are indicated by name and sample type. Exon-intron structures for RefSeq genes involved in the fusions are indicated in each panel in blue. Asterisks indicate samples which contain the fusions similar to those described in the main text.
